# Cardiovascular complications after common bile duct stone extractions

**DOI:** 10.1007/s00464-020-07766-3

**Published:** 2020-07-01

**Authors:** Eva-Lena Syrén, Lars Enochsson, Staffan Eriksson, Arne Eklund, Bengt Isaksson, Gabriel Sandblom

**Affiliations:** 1 Department of Surgical Sciences, Uppsala University, 751 35 Uppsala, Sweden; 2 Centre for Clinical Research, Västmanland Hospital, Västerås, Sweden; 3 Department of Surgical and Perioperative Sciences, Sunderby Research Unit, Umeå University, SurgeryUmeå, Sweden; 4 Department of Clinical Science and Education Södersjukhuset, Karolinska Institute, Stockholm, Sweden; 5 Department of Surgery, Södersjukhuset, Stockholm, Sweden

**Keywords:** ERCP, Choledocholithiasis, Cardiovascular complication

## Abstract

**Background:**

Common bile duct stone (CBDS) is a common condition the rate of which increases with age. Decision to treat in particular elderly and frail patients with CBDS is often complex and requires careful assessment of the risk for treatment-related cardiovascular complications. The aim of this study was to compare the rate of postoperative cardiovascular events in CBDS patients treated with the following: ERCP only; cholecystectomy only; cholecystectomy followed by delayed ERCP; cholecystectomy together with ERCP; or ERCP followed by delayed cholecystectomy.

**Methods:**

The study was based on data from procedures for gallstone disease registered in the Swedish National Quality Register for Cholecystectomy and Endoscopic Retrograde Cholangiopancreatography (GallRiks) 2006–2014. ERCP and cholecystectomy procedures performed for confirmed or suspected CBDS were included. Postoperative events were registered by cross-matching GallRiks with the National Patient Register (NPR). A postoperative cardiovascular event was defined as an ICD-code in the discharge notes indicating myocardial infarct, pulmonary embolism or cerebrovascular disease within 30 days after surgery. In cases where a patient had undergone ERCP and cholecystectomy on separate occasions, the 30-day interval was timed from the first intervention.

**Results:**

A total of 23,591 underwent ERCP or cholecystectomy for CBDS during the study period. A postoperative cardiovascular event was registered in 164 patients and death within 30 days in 225 patients. In univariable analysis, adverse cardiovascular event and death within 30 days were more frequent in patients who underwent primary ERCP (*p* < 0.05). In multivariable analysis, adjusting for history of cardiovascular disease or events, neither risk for cardiovascular complication nor death within 30 days remained statistically significant in the ERCP group.

**Conclusions:**

Primary ERCP as well as cholecystectomy may be performed for CBDS with acceptable safety. More studies are required to provide reliable guidelines for the management of CBDS.

Common bile duct stone (CBDS) is a common disease with varying clinical manifestations. CBD stones are often asymptomatic, but may cause biliary pancreatitis, obstructive jaundice, cholangitis, or recurrent pain [1, 2]. There are several accepted methods of treatment for CBDS [3–8]. Cholecystectomy with or without concomitant intraoperative rendezvous endoscopic retrograde cholangiopancreaticography (ERCP), if CBDS is found on intraoperative cholangiography (IOC), is a well-established, safe and cost-effective method for patients considered fit for surgery [9–12].

ERCP is sometimes performed as part of a two-stage procedure, either as ERCP followed by delayed cholecystectomy or cholecystectomy followed by delayed ERCP [1, 13]. In some patients, where high age and comorbidity render them too high risk for surgery, ERCP with sphincterotomy and stone extraction may be preferred as sole intervention, even if recurrent choledocholithiasis is more common when ERCP is the only treatment performed [14]. If ERCP is performed without the aid of antegrade introduction of a guidewire at IOC, 4–18% of attempts fail due to inability to cannulate the bile duct [9]. Surgical complications, especially post-ERCP pancreatitis (PEP), are also more frequent after standard ERCP compared to rendezvous ERCP [10, 11, 15–17].

The frequency of CBDS increases with age. This complicates management as comorbidity and frailty increase the risk for intervention-related complications. Cardiovascular disease and biliary stone disease share risk factors such as obesity, hypertension, diabetes, dyslipidemia, and cigarette smoking [18–20]. There also appears to be an association between gallstone disease and cardiovascular disease [21].

The cardiovascular complication and pulmonary thromboembolism (PTE) rates following laparoscopic cholecystectomy and ERCP are low, even in elderly patients (< 2%) [22–25].

The aim of this study was to compare postoperative cardiovascular complication rates (myocardial infarct, pulmonary thromboembolism and/or cerebrovascular disease) in patients with CBDS treated with: ERCP only; cholecystectomy only; cholecystectomy followed by delayed ERCP; cholecystectomy combined with ERCP; or ERCP followed by delayed cholecystectomy.

## Materials and methods

This study was based on procedures for gallstone disease registered in the Swedish National Quality Register for Cholecystectomy and Endoscopic Retrograde Cholangiopancreatography (GallRiks) 2006–2014. GallRiks registration began 1st May 2005 and now covers approximately 90% of all cholecystectomies and ERCPs performed in Sweden, including patient- and procedure-related data. All intra- and postoperative adverse events, including cardiovascular complications, are registered, and the completeness of 30-day follow-up of postoperative complications is approximately 95%. GallRiks is regularly externally validated [26, 27].

In the present study, ERCP as well as cholecystectomy performed with confirmed or suspected CBDS as indication were included. Patients who underwent cholecystectomy combined with ERCP were also included as long as either of the procedures was performed with CBDS as indication. ERCP or cholecystectomy performed because of malignant stricture or suspicion of cancer were excluded as well as patients who underwent one or more procedures without CBDS as indication.

Patients with confirmed or suspected CBDS were divided into five treatment groups: ERCP only; cholecystectomy only; cholecystectomy followed by delayed ERCP, cholecystectomy combined with ERCP; or ERCP followed by delayed cholecystectomy.

Postoperative events were registered by cross-matching GallRiks with the National Patient Register (NPR). Data on cardiovascular complications within 30 days after surgery, defined as a diagnosis in the discharge notes with an ICD-code indicating myocardial infarct, pulmonary embolism or cerebrovascular disease (not including those who had an ICD-code indicating cerebrovascular disease prior to surgery), were retrieved from the NPR. If a patient had undergone both ERCP and cholecystectomy, the 30-day interval was timed from the first intervention. Data on previous cardiovascular events were also obtained from the NPR.

The Regional Ethics Review Board in Stockholm approved the study 18th March 2015 (IRB-approval, reference number: 2015/339-31/1).

Consent from the patient to participate in register-based research is required for registration in GallRiks. Patients are given the opportunity to withdraw all their personal data at any time from the register.

## Statistics

In order to adjust for confounders, multivariate logistic regression analyses were performed, with cardiovascular event (myocardial infarct and/or pulmonary embolus and/or cerebrovascular disease) and death within 30 days as endpoints. The multivariate models were based on age (≥ 80 years vs < 80 years), ASA score (III–V vs I–II), gender, treatment and history of cardiovascular condition or event (myocardial infarct, heart failure, peripheral vascular disease, cerebrovascular event, diabetes with secondary complication or pulmonary embolism). Patients who underwent cholecystectomy and ERCP during the same procedure and those who underwent cholecystectomy and delayed ERCP were grouped together with the cholecystectomy group, whereas those who underwent ERCP and delayed cholecystectomy were grouped together with the ERCP group. This grouping was based on which procedure was the primary intervention aimed at managing the CBDS.

Poisson regression was used to calculate the 30-day age- and gender-adjusted standardized mortality ratio (SMR) based on the expected mortality rate extrapolated from the Swedish general population in 2007.

## Results

**Fig. 1:**
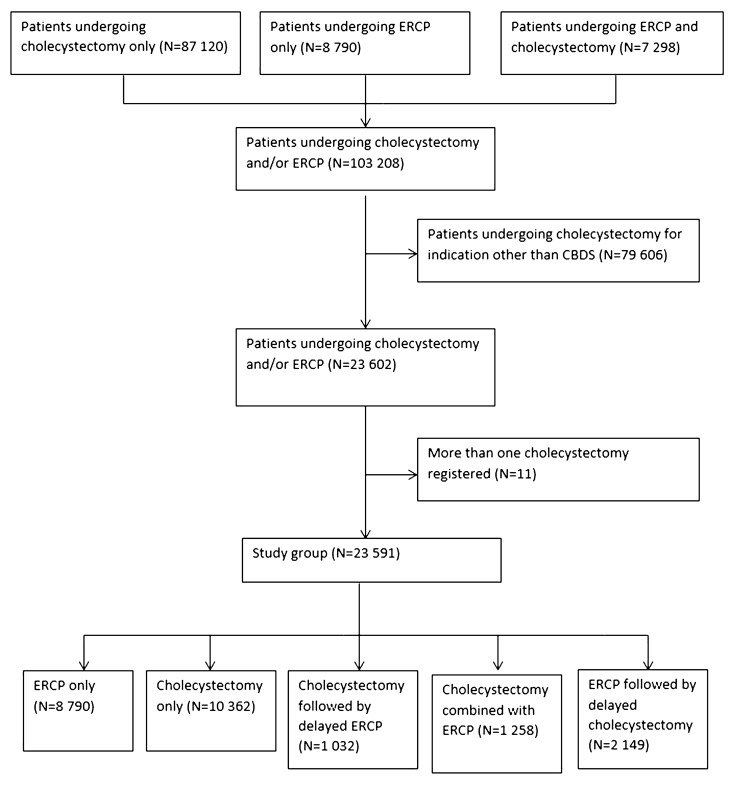
Flow chart. Confirmed or suspected CBDS as indication for treatment

During the study period, 103,208 patients underwent cholecystectomy and/or ERCP due to gallstone disease. After excluding cholecystectomies performed without preoperatively diagnosed common bile duct stone and patients registered with more than one cholecystectomy, 23,591 patients remained in the study group. Of those, 8790 underwent ERCP only, 10,362 cholecystectomy only, 1032 cholecystectomy followed by delayed ERCP, 1258 cholecystectomy combined with ERCP, and 2149 ERCP followed by delayed cholecystectomy (Fig. 1).

Patients in the ERCP only group were older, more often female and ASA grade III–V vs I–I compared to the cholecystectomy only group. A previous history of cardiovascular disease (myocardial infarct, heart failure, peripheral vascular disease, cerebrovascular event, diabetes with secondary complication or pulmonary embolism) was also much more common in the ERCP only group. In the group ERCP followed by delayed cholecystectomy, patients were older and more often had a previous history of cardiovascular disease compared to patients in the groups cholecystectomy combined with ERCP, and cholecystectomy followed by delayed ERCP (Table 1).

**Table 1:** Baseline characteristics

	ERCP only (*N* = 8790)	Cholecystectomy only (*N* = 10,362)	Cholecystectomy followed by delayed ERCP (*N* = 1 032)	Cholecystectomy combined with ERCP (*N* = 1258)	ERCP followed by delayed cholecystectomy (*N* = 2149)
*Gender*					
Men	3653 (36.1%)	4650 (46.0%)	413 (4.1%)	479 (4.7%)	918 (9.1%)
Women	5137 (38.1%)	5712 (42.4%)	619 (4.6%)	779 (5.8%)	1231 (9.1%)
*Mean age, years (standard deviation)*	73.5 (15.5)	53.5 (17.8)	55.0 (17.9)	49.4 (18.4)	58.8 (16.1)
*ASA*					
I	1583 (18.9%)	4631 (56.1%)	429 (5.2%)	646 (7.8%)	980 (11.9%)
II	4627 (41.5%)	4570 (41.0%)	468 (4.2%)	515 (4.6%)	985 (8.7%)
III	2451 (61.9%)	1092 (27.8%)	128 (3.2%)	95 (2.4%)	191 (4.8%)
IV	148 (62.7%)	66 (28.0%)	7 (3.0%)	2 (0.8%)	13 (5.5%)
V	1 (25.0%)	3 (75.0%)	0	0	0
*History of cardiovascular disease and events*					
Myocardial infarct	1140 (13.0%)	373 (3.6%)	49 (4.7%)	35 (2.8%)	103 (4.8%)
Cardiac failure	1406 (16.0%)	363 (3.5%)	43 (4.2%)	34 (2.7%)	100 (4.7%)
Peripheral vascular disease	691 (7.9%)	209 (2.0%)	19 (1.8%)	26 (2.1%)	72 (3.4%)
Cerebrovascular event	1536 (17.5%)	512 (4.9%)	54 (5.2%)	54 (4.3%)	130 (6.0%)
Diabetes with secondary complication	454 (5.2%)	188 (1.8%)	21 (2.0%)	14 (1.1%)	53 (2.5%)
Pulmonary embolism	270 (3.1%)	112 (1.1%)	16 (1.6%)	7 (0.6%)	26 (1.2%)

In all, a postoperative cardiovascular event was registered in 164 cases and death within 30 days in 225 cases. Postoperative adverse event and death within 30 days were more frequently seen in the ERCP only group compared to the other groups. Myocardial infarct was at least twice as common (0.71%) and cerebrovascular lesion at least three times as common (0.26%) in the ERCP only group compared to the other groups. The incidence of pulmonary embolism was more equally distributed between groups and most common in the group cholecystectomy followed by delayed ERCP (0.39%). Postoperative death within 30 days was between 5 and 20 times more common in the ERCP only group (1.97%) (Table 2).

**Table 2:** Postoperative adverse events in confirmed or suspected CBDS within 30 days in the Swedish National Quality Register for Cholecystectomy and Endoscopic Retrograde Cholangiopancreatography (GallRiks) 2006–2014

	ERCP only (*N* = 8790)	Cholecystectomy only (*N* = 10,362)	Cholecystectomy followed by delayed ERCP (*N* = 1032)	Cholecystectomy combined with ERCP (*N* = 1258)	ERCP followed by delayed cholecystectomy (*N* = 2149)	Total complication incidence (*N* = 164) and death (*N* = 225)
Myocardial infarct	62 (0.71%)	13 (0.13%)	3 (0.29%)	1 (0.08%)	3 (0.14%)	82
Cerebrovascular lesion	23 (0.26%)	5 (0.05%)	1 (0.10%)	0 (0%)	2 (0.09%)	31
Pulmonary embolism	23 (0.26%)	19 (0.18%)	4 (0.39%)	3 (0.24%)	2 (0.09%)	51
Postoperative death	173 (1.97%)	43 (0.41%)	4 (0.39%)	3 (0.24%)	2 (0.09%)	225

Age ≥ 80 years, ASA > 1 and history of cardiovascular disease or event were all risk factors for postoperative complication and death. In the univariable and multivariable logistic regression analyses, cardiovascular complication and death within 30 days were studied in the ERCP group (ERCP only + ERCP with delayed cholecystectomy), with the cholecystectomy group as reference (cholecystectomy with or without combined ERCP + cholecystectomy with delayed ERCP). In the univariable analysis, adverse cardiovascular event (OR 2.74, 95% confidence interval [CI] 1.95–3.84, *p* < 0.001) and death (OR 4.10, CI 3.00–5.62, *p* < 0.001) were more frequent in the ERCP group. In the multivariable analysis, adjusting for history of cardiovascular conditions or events, neither the risk for cardiovascular complication (OR 1.12, CI 0.77–1.64, *p* < 0.548) nor death within 30 days (OR 1.38, CI 0.97–1.96, *p* < 0.071) remained statistically significant in the ERCP group (Table 3).

**Table 3:** Univariable and multivariable analyses of factors predicting cardiovascular event and death within 30 days after surgical and/or endoscopic treatment for confirmed or suspected CBDS in the Swedish National Quality Register for Cholecystectomy and Endoscopic Retrograde Cholangiopancreatography (GallRiks) 2006–2014

Univariable	Cardiovascular complication		Death	
	Odds ratio (95% confidence interval)	*p*	Odds ratio (95% confidence interval)	*p*
Age ≥ 80 years (ref < 80 years)	4.37 (3.20–5.60)	< 0.001	9.60 (7.20–12.79)	< 0.001
Men (ref women)	1.16 (0.85–1.59)	0.340	1.19 (0.91–1.55)	0.197
*ASA I *(*ref*)				
ASA II	3.83 (2.16–6.79)	< 0.001	6.42 (3.08–13.35)	< 0.001
ASA III	9.82 (5.51–17.52)	< 0.001	31.39 (15.32–64.31)	< 0.001
ASA IV	26.03 (11.44–59.22)	< 0.001	150.02 (67.94–331.23)	< 0.001
ASA V	–	–	343.38 (32.20–3662.14)	< 0.001
History of cardiovascular disease or event^a^	10.20 (7.12–14.60)	< 0.001	6.25 (4.74–8.23)	< 0.001
ERCP (ref cholecystectomy)^b^	2.74 (1.95–3.84)	< 0.001	4.10 (3.00–5.62)	< 0.001

Multivariable	Cardiovascular complication		Death	
ERCP (ref cholecystectomy)^a^	1.12 (0.77–1.64)	0.548	1.38 (0.97–1.96)	0.071

^a^History of myocardial infarct, heart failure, peripheral vascular disease, cerebrovascular event, diabetes with secondary complication or pulmonary embolism

^b^In cases where ERCP as well as cholecystectomy were performed, allocation was determined by the primary procedure. If cholecystectomy and ERCP were performed as one procedure, the procedure was allocated to the cholecystectomy group

## Discussion

In this register-based study, we analyzed postoperative cardiovascular complications in a large number of patients who underwent surgical treatment for confirmed or suspected CBDS. The study was based on prospectively assembled population-based data from GallRiks covering a long period of time. The study focused on the most common postoperative cardiovascular events *i.e.* myocardial infarct and/or pulmonary embolus and/or cerebrovascular disease as well as death within 30 days. Although the study could not show any approach to be safer than the others, our results may help in future treatment-decisions.

We decided to focus on and present only the incidence of cardiovascular complications. There are differences in the burden of cardiovascular disease between Sweden and other parts of the world. U.S. and Swedish data diverge to a lesser extent than what may be seen when the Western World is compared to areas outside Western Europe and North America [28].

The prevalence of gallstone-related symptoms, including CBDS, in the population is high (7–15%) and high age is a significant risk factor for prolonged hospital stay and death after any procedure for gallstone removal [29, 30]. The comorbidity rate in elderly patients undergoing treatment for choledocholithiasis is high compared to younger patients [31]. Frailty is a crucial risk factor, although it is difficult to quantify. We consider age as surrogate measure for frailty, although age and frailty only partly correlate.

Tobacco use and obesity are major risk factors that have to be taken into account when estimating the risk for cardiovascular complications following a surgical or endoscopic intervention. Even if smoking and BMI are included in the ASA physical status they were not registered routinely in GallRiks during the period of the study [32]. We also lack data on medications, including anticoagulation. There was no consistent predetermined national algorithm administration during the period of study. In Sweden the prevailing routine is to interrupt anticoagulation therapy before surgery and ERCP and restart anticoagulation postoperatively, but each hospital follow their own local guidelines.

Anesthesia was not included as predictor in the present study. It has though been explored in a recent study based on GallRiks data which has shown more post-procedural complications occurred after ERCPs performed under deep sedation compared to those performed under general anesthesia [33].

The five treatment groups in this study are not predetermined and the affiliation to a certain group is dependent on several heterogeneous factors such as complexity and status of the biliary disease and preference of the deciding doctor or local treatment regimes. Several strategies are employed to manage CBDS disease, and methods and timing vary from hospital to hospital [3]. Even if cholecystectomy combined with rendezvous ERCP is standard in many departments, the decision on which treatment is used for CBDS is usually based on local tradition and ERCP-competence. Before the introduction of intraoperative rendezvous ERCP, it was common that patients with CBDS were treated with a two-stage procedure, either preoperative ERCP followed by cholecystectomy or cholecystectomy followed by postoperative ERCP [34]. There are still units where cholecystectomy is performed on regular basis but where there is a lack of ERCP resources and a two-stage procedure thus remains the only choice [34].

Even if early cholecystectomy appears to be safe in elderly, there is a tendency to choose minimally invasive treatment methods such as ERCP when it comes to older, frail patients with comorbidity [35]. No subsequent cholecystectomy was registered for any of the 8790 patients with ERCP as sole intervention. It is, however, possible that some of the patients underwent cholecystectomy after the period of the study. As a cholecystectomy at that late state could not be expected to be performed with the aim of preventing CBDS, we do not think that they are relevant for the aims of the present study.

It is possible that procedure-related complications preceded the cardiovascular complications, which also has to be taken into account when deciding on treatment of common bile duct stones. Even if we believe that most complications are included, it can’t be excluded that registration of some adverse events could have been missed in the analysis regarding those patients who underwent both ERCP and cholecystectomy as two separate interventions and with a long interval between procedures.

In this study patients who were selected for ERCP were older and had more comorbidity than patients in the other treatment groups. Myocardial infarction, cardiac failure, peripheral vascular disease, cerebrovascular event, diabetes with secondary complication and pulmonary embolism were strong predictors for cardiovascular complication and death after surgical treatment for CBDS. We believe that the selection of frail patients and patients with greater comorbidity for ERCP explains why ERCP was significant in univariate analysis. In multivariable analysis, adjusting for history of cardiovascular disease or events, neither risk for cardiovascular complication nor death within 30 days remained statistically significant in the ERCP group.

Based on the results of this study we believe both ERCP as well as cholecystectomy may be used for CBDS treatment in the elderly with acceptable safety.
